# Aplastic Anemia and Chagas Disease: *T. cruzi* Parasitemia Monitoring by Quantitative PCR and Preemptive Antiparasitic Therapy

**DOI:** 10.3390/tropicalmed7100268

**Published:** 2022-09-27

**Authors:** Noêmia Barbosa Carvalho, Vera Teixeira de Freitas, Rita Cristina Bezerra, Erika Shimoda Nakanishi, Elvira Pereira Velloso, Hermes Ryoiti Higashino, Marjorie Vieira Batista, Guilherme Henrique Fonseca, Vanderson Rocha, Silvia Figueiredo Costa, Maria Aparecida Shikanai-Yasuda

**Affiliations:** 1 Division of Infectious and Parasitic Diseases, Hospital das Clínicas, Faculdade de Medicina, University of São Paulo (HC-FMUSP), Av. Enéias C. Aguiar, 255, Sao Paulo 05403000, Brazil; 2 Department of Infectious and Parasitic Diseases, Faculdade Medicina, University of Sao Paulo (FMUSP), Av. Enéias C. Aguiar, 470, Sao Paulo 05403000, Brazil; 3 Laboratory of Medical Investigation in Immunology (LIM 48), Hospital das Clínicas, Faculdade de Medicina, University of Sao Paulo, Av. Enéias C. Aguiar, 470, Sao Paulo 05403000, Brazil; 4 Laboratory of Medical Investigation in Parasitology (LIM 46), Av. Enéias C. Aguiar, 470, Sao Paulo 05403000, Brazil; 5 Service of Hematology, Transfusion and Cell Therapy and Laboratory of Medical Investigation in Pathogenesis and Directed Therapy in Onco-Immuno-Hematology (LIM-31), Hospital das Clínicas, Faculdade de Medicina, University of Sao Paulo, Av. Enéias C. Aguiar, 255, Sao Paulo 05403000, Brazil; 6 Churchill Hospital, Oxford University, Old Rd, Headington, Oxford OX3 7LE, UK; 7 Laboratory of Medical Investigation in Protozoology (LIM 49), Hospital das Clínicas, Faculdade de Medicina, University of São Paulo, Av. Enéias C. Aguiar, 470, Sao Paulo 05403000, Brazil

**Keywords:** Chagas disease, aplastic anemia, Chagas disease plus aplastic anemia, qPCR for *T. cruzi* parasitemia monitoring, preemptive antiparasitic therapy

## Abstract

**Background:** Aplastic anemia is a rare and life-threatening condition, seldomly witnessed concomitantly with Chagas disease. We aim to discuss the management of these patients under risk of chronic Chagas disease reactivation (CDR), a severe condition with a high morbimortality that occurs in chronic Chagas disease patients under immunosuppression. **Case reports:** *Trypanosoma cruzi* (*T. cruzi*) parasitemia was monitored in three patients for 4–58 months by conventional PCR (cPCR), quantitative PCR (qPCR), microhematocrit/buffy coat, blood culture, and/or xenodiagnosis. One patient received antiparasitic treatment (benznidazole) and the other received allopurinol. Although parasitemia was controlled during and after benznidazole treatment at 300 mg/d for 51 days, in one patient, hematologic parameters worsened continuously before, during, and after treatment. Allopurinol led only to the temporary suppression of *T. cruzi* parasitemia in the second patient, but after danazol and hematological improvement, parasitemia became undetectable until the end of monitoring. **Discussion and Conclusion**: Unexpected undetectable or low parasitemia by cPCR/qPCR was reported. We show that the monitoring of parasitemia by qPCR and the use of preemptive therapy when the parasitemia was positive proved to be beneficial to our patients. As a result of the toxicity of more effective antiparasitics, shorter regimens of benznidazole or less toxic drugs in preemptive therapy are options that deserve future studies.

## 1. Introduction 

Acquired aplastic anemia (AA) is a rare, life-threatening condition characterized by cytopenia and bone marrow (BM) failure, caused by a physical, chemical, or biological factors [1]. Bone marrow hypocellularity has been attributed to the immune aggression of cytotoxic T cells towards hematopoietic cells [2]. An increased number of Th17 cytotoxic cells (CD3+, CD4+, and IL-17) has been shown in peripheral blood along with a decrease in regulatory T cells (CD4+, CD25, high CD127, and low FoxP3) [3]. Mortality is above 80% at two years, but survival has improved a lot with immunosuppressive therapy and hematopoietic stem cell transplantation (HSCT). Standard immunosuppressive therapy consists of anti-thymocyte globulin (ATG) and cyclosporine A and has been recently associated with eltrombopag [4].

Recovery after treatment with anti-thymocyte globulin (ATG), cyclosporin A, or anti-lymphocyte globulin, suggests that autoimmunity may be involved in the pathogenesis of AA. Immune dysfunction is expressed by an altered number of lymphocyte subsets, increased effects of cytotoxic lymphocytes on hematopoietic stem and progenitor cells (HSPC), and low percentages of NK cells. In parallel, genetic background and intrinsic deficiencies of the HSPC, evidenced by somatic mutations such as shortened telomeres and viral infection, may also be associated with AA pathogenesis [1,5].

Although Chagas disease is endemic in Latin America, aplastic anemia in patients bearing chronic Chagas disease has rarely been reported [6]. The immune dysfunction combined with immunosuppressive therapy in AA patients poses a risk for chronic Chagas disease reactivation (CDR). In addition, HSCT and immunosuppressive therapy for graft-versus-host disease (GVHD) increase the risk of this reactivation by inducing changes in the balance of the host–parasite interaction that controls parasite growth. To the best of our knowledge, AA plus chronic Chagas disease under immunosuppression has been published once in a patient who had Chagas disease reactivation (CDR) after allogeneic HSCT [6].

Chagas disease is a neglected tropical disease affecting approximately six million people in Latin America, and is now globally widespread due to the immigration of infected people from Latin America to other continents [7]. It is caused by the protozoan *T. cruzi* and is acquired through vector and maternal–fetal transmissions, blood product transfusions, organ transplantations, contaminated food, and accidents with infected biological material [8]. After contamination by a triatomine bug, one in ten infected individuals develop an acute apparent disease, characterized by high parasitemia and fever, bilateral periorbital edema, and/or an infectious mononucleosis-like syndrome, and, more rarely, myocarditis and encephalitis; however, most infected individuals are oligosymptomatic or asymptomatic. The disease evolves in 6 to 12 weeks to the chronic phase, with low parasitemia, which is only detectable by molecular and parasitological enrichment methods [8]. In this phase, an “indeterminate form” represents 70% of cases, without symptoms or apparent heart or digestive involvement; chronic chagasic cardiomyopathy, approximately 20% of cases; digestive involvement, 10% of cases; and mixed cardiac plus digestive form, 5% of cases [8]. 

Chronic Chagas disease patients under immunosuppression and/or immune dysfunction caused by aplastic anemia are at risk for CDR characterized by severe morbimortality [9,10]. 

Screening for Chagas disease is mandatory in patients living in endemic regions, for those born from infected mothers, or for those that received multiple blood or derivatives transfusions. Two serological tests with different methodologies confirm the diagnosis of *T. cruzi* infection. For allogeneic HSCT, infected donors are not accepted, except in emergencies, where no other non-*T. cruzi* infected donor is available. Prospective monitoring of parasitemia plays a key role in its control and in reducing the risk of CDR in immunosuppressed patients either with HIV infection [11,12], autoimmune diseases [13], or undergoing transplantation [14,15].

In this scenario of AA associated with chronic CD, the evolution of both diseases and the risk of CDR is not known, representing challenges for patients’ follow-up. An additional issue to address is the myelotoxicity and other adverse events of benznidazole and nifurtimox, both recommended for antiparasitic treatment [8] and compulsory for acute Chagas disease and CDR.

This work describes the role of *T. cruzi* parasitemia monitoring in the management of three AA patients with chronic Chagas disease, aiming to analyze the role of qPCR and preemptive therapy to prevent CDR. 

## 2. Material and Methods

The inclusion criterion for Chagas disease was reactivity by two different serologies (ELISA and Indirect Immunofluorescence) [11]. Parasitemia by *T. cruzi* was monitored by cPCR using the S35/S36 primer pair and qPCR for the microsatellite sequence TCZ3/TCZ4, as previously described [9]; blood culture; and/or xenodiagnosis [8]. Chagas disease reactivation was checked by the microhematocrit or buffy coat in the peripheral blood [8]. 

### Ethical Approval

This study was approved by the Ethics and Research Committee of the Hospital das Clínicas da Faculdade de Medicina, University of São Paulo, Brazil (protocol number 095/1995); and the inclusion of retrospectively collected data was approved by the Ethics and Research Committee of the Department of Infectious and Parasitic Diseases of the Faculdade de Medicina, University of São Paulo, under protocol 003/22, ensuring that the confidentiality of patients’ identity was maintained. All data were collected anonymously from medical records.

## 3. Case Reports

Patient 1

Male, 55 years old, from the state of Alagoas, Brazil, with no previous antiparasitic treatment. Underwent *T. cruzi* parasitemia monitoring for 28 months.

Previous arterial hypertension and Chagas heart disease with a pacemaker due to total atrioventricular block and ventricular dysfunction.

On 29 January 2019, the patient was admitted to the emergency room with symptoms of presyncope, gingival bleeding, and bruising. Hb = 4.4 g/dL; reticulocytes, 7000/mm^3^; platelets 8000/mm^3^; total leukocytes 2980/mm^3^ (neutrophils 760/mm^3^). Bone marrow biopsy (30 January 2019) showed global hypocellularity (10%). A diagnosis of severe aplastic anemia was made, and cyclosporine use began in February.

He was hospitalized in late March for 15 days due to low-risk febrile neutropenia with clinical response. Because of possible ATG administration, *T. cruzi* parasitemia was performed. Positive cPCR and qPCR below detection levels were observed on 27 March 2019 (Table 1), and preemptive therapy with Benznidazole for 60 days was suggested. He received 300 mg/day of benznidazole starting on 08 April 2019, for 51 days, before ATG, which was scheduled for infusion if *T. cruzi* PCR was negative on day 30 of benznidazole. 

On 30 April 2019, the complete blood count (CBC) showed Hb 8.4 g/dL; platelets 5000/mm^3^; leukocytes 2430/mm^3^ (neutrophils 340/mm^3^, lymphocytes 1880/mm^3^, and monocytes 210/mm^3^).

The patient’s evaluation on the eighth day of benznidazole revealed a good response; on the 15th day, cPCR was negative, as well as on the 30th day (Table 1). On day 45, he reported mild epigastralgia. 

On 27 May 2019, BCC showed Hb 5.6 g/dL; Ht 16.1%; 1000 platelets/mm^3^; leukocytes 1210/mm^3^ (neutrophils 280/mm^3^, lymphocytes 730/mm^3^, and monocytes 200/mm^3^). 

During the period from 6 February 2019 to 11 March 2020, he received 200 mg/day of cyclosporine, tranexamic acid, losartan, atenolol, multiple red blood cell transfusions, and platelet concentrates by apheresis.

ATG infusion was prescribed from 29 May 2019 to 2 June 2019, and prednisone prescription followed the protocol.

On 10 December 2019, a new bone marrow biopsy was performed and showed no improvement in cellularity. Cyclosporine was suspended on 11 March 2020, and Alemtuzumab was introduced from 2 March 2020 to 6 March 2020, with total doses of 100 mg.

Direct examination of parasitemia was persistently negative during 26 months of follow-up and after ATG; cPCR was continuously negative after the treatment, and qPCR was negative on 21 July 2021 (Table 1).

He underwent courses of antibiotics associated with neutropenia and multiple transfusions for symptomatic anemia and cutaneous–mucosal bleeding associated with thrombocytopenia. Hospitalization on 5 July 2021, for febrile neutropenia due to pulmonary infection, with clinical improvement.

Hematological examinations from this period up until 30 July 2021 revealed Hb from 5.1-7.2 g/dL, leukocytes from 160–280/mm^3^, and platelets from 8000–47,000/mm^3^.

On 31 July 2021, he evolved with a refractory septic shock of abdominal focus (neutropenic enterocolitis) and died on 2 August 2021.

Patient 2

Female, 47 years old, born in Bolivia. Underwent *T. cruzi* parasitemia monitoring for 4 months and 20 days.

She came to Brazil for treatment for aplastic anemia diagnosed in 2014 and was previously treated in Bolivia. She had Chagas disease with non-typical cardiac alteration (ECG: left axis deviation, echocardiogram, myocardial hypertrophy with preserved systolic function, and moderate diastolic dysfunction), morbid obesity, and arterial hypertension in treatment.

On 20 May 2016, she was hospitalized with pancytopenia, symptomatic anemia, and thrombocytopenia with gingival and vaginal bleeding. She was admitted again by febrile neutropenia 1.5 months later, which resolved after clinical treatment. Mycophenolate mofetil (360 mg 12/12 h) was reintroduced and she required multiple transfusions.

*T. cruzi* parasitemia was undetectable by both molecular methods and direct microscopy for CDR diagnosis in this period (Table 2).

On 8 June 2016, she was started on cyclosporine (200 mg/d) after performing an aspirate and BM biopsy that confirmed AA. On 15 July 2016, ATG was administered for five days at a dose of 2.5 mg/kg/day, and *T. cruzi* parasitemia was negative even after ATG (Table 2).

Four months later, under the daily use of cyclosporine, no hematological response was observed. Cyclosporine was suspended after a pulmonary infection associated with febrile neutropenia. 

On 1 February 2017, without an HLA-matched donor or improvement in peripheral blood after seven months of ATG, danazol was introduced at a dose of 400 mg/day.

Ten days later, she was admitted to the emergency unit due to septic shock of abdominal focus/enterocolitis, evolving to death on 13 February 2017.

There was never a detection of positive parasitemia during the 140 days of parasitemia monitoring, so antiparasitic treatment was not administered.

Patient 3

Female, 63 years old, born in Bolivia, living in Brazil since 2011. Underwent *T. cruzi* parasitemia monitoring for 58 months.

Comorbidities: chronic hepatitis B (tenofovir 300 mg/d), HA (losartan, amlodipine), and indeterminate form of Chagas Disease; denied antiparasitic treatment.

On September 2011, she had fatigue and epistaxis. Bone marrow aspiration and biopsy showed a global hypocellularity, diagnosed with bone marrow aplasia.

On 9 November 2011, CBC showed Hb 7.6 g/dL; platelets 18,000/mm^3^; leukocytes 2000/mm^3^ (neutrophils 400/mm^3^, lymphocytes 1500/mm^3^, and monocytes 100/mm^3^). 

Upon diagnosis of severe aplastic anemia, she started treatment on 14 December 2011, at the Hematology Unit with 200 mg/day of cyclosporine, with no response at three months and partial response at six months.

Positive cPCR and qPCR (9.32 parasites Eq/mL) and negative direct microscopic examination, with no reactivation symptoms, were shown three months after the initiation of cyclosporine; then, negative cPCR and qPCR below the detection level, and positive blood culture were found (Table 1). Preemptive treatment for Chagas disease due to intermittent parasitemia in the presence of immunosuppression was discussed. In September 2012, positive cPCR and qPCR = 4.5 parasites Eq/mL, positive blood culture, and negative direct microscopic examination for *T. cruzi* were found (Table 1).

In the absence of reactivation criteria, antiparasitic therapy with a lower risk of myelotoxicity was considered. The risk–benefit of benznidazole was evaluated, as well as the possibility of treatment with posaconazole, which was currently unavailable, and allopurinol was opted for as an alternative drug.

On 9 January 2013, cyclosporine was replaced due to an adverse effect, and danazol was introduced at a dose of 300 mg/day.

Allopurinol was started on 23 May 2013, at a dose of 300 mg/day for the first 14 days, then reduced to 200 mg/day (dose adjusted for creatinine clearance), completing a total of 38 days (non-complete treatment). After treatment, parasitemia occurred (Table 2).

On 9 April 2014, a complete response to danazol was observed and was withdrawn after one year of a plateau response. Danazol was suspended on 18 March 2015. The patient was stable, without infectious complications and bleeding or symptomatic anemia.

On 28 March 2014, CBC showed Hb 11.3 g/dL; platelets 200,000/mm^3^; leukocytes 4150/mm^3^ (neutrophils 2490/mm^3^, lymphocytes 1300/mm^3^, and monocytes 290/mm^3^). 

During the period of stability of the underlying disease, she evolved with positive cPCR and undetectable qPCR for 15 months, but in the final 14 months of follow-up, cPCR reverted to negative and qPCR remained undetectable (Table 2).

## 4. Discussion 

Two patients showed severe aplastic anemia and received an aggressive immunosuppressive regimen (cyclosporine, ATG, and corticosteroid with or without alemtuzumab), consequently presenting a deficiency of T lymphocytes and macrophage functions. One patient with moderate aplastic anemia received cyclosporine and danazol. The crucial role of CD4+, macrophage activation, and IFNγ secretion to kill the parasites has been shown [16,17,18]. Interferon-gamma controls *T. cruzi* growth in synergism with TNFα, through nitrite oxide secretion [19].

As seen in Table 1 and Table 2, lymphopenia did not seem to be a relevant factor associated with positive parasitemia in our patients (P1 and P3) as it was concomitant with a low and/or a normal number of lymphocytes/mm^3^. In addition, positive parasitemia does not seem to depend on the absolute number of granulocytes or monocytes.

The unexpected undetectable and/or low levels of parasitemia in our three patients raised questions concerning the control of parasite growth. For example, P2 never had detectable parasitemia in the presence of a low/normal number of lymphocytes/mm^3^ concomitantly with a low number of neutrophils and platelets under different drugs. 

Unfortunately, lymphocyte or macrophage functions, as well as lymphocyte subsets or cytokines were not analyzed, except in P1, with low levels of CD4 (CD4/CD8 = 0.6 and CD4/CD8 = 0.18) concomitantly with < 100 lymphocytes/mm^3^ and negative parasitemia. 

As already described, before immunosuppressive treatment, AA patients may have normal to hyperactive immune systems and intact intertegumentary and mucosal barriers [20], but they progress with frequent bacterial and fungal infections under immunosuppressive therapy. The deaths of P1 and P2 were associated with bacterial infections and neutropenia <500 cells/mm^3^ [20,21,22], but mycobacterial, *T. cruzi,* and other intracellular infections have rarely been described in AA patients [6,20]. Considering the possible absence of adaptative anti-*T. cruzi* immunity, other expressions of immune responses, such as an innate response, could control parasite growth and need to be better understood.

Another question to be addressed is the choice for antiparasitic treatment, either efficient but myelotoxic drugs (benznidazole and nifurtimox) [8], or the less toxic and less efficient drug, allopurinol [23,24,25].

Benznidazole was prescribed to P1 before ATG treatment as a result of positive parasitemia and was interrupted earlier than the classic period of 60 days. Decreasing Hb, leucocytes, and platelets were seen during antiparasitic therapy; however, besides possible myelotoxicity, the severe natural evolution of AA needs to be considered. From the beginning of antiparasitic therapy to 28 months later, parasitemia was negative, even though ATG, cyclosporine, corticosteroid, and alemtuzumab had been prescribed. According to guidelines, a regimen of 5 mg/kg/day of BNZ for 60 days is mandatory to treat CDR and acute Chagas disease [8], so in AA patients on ATG, careful monitoring is necessary to control parasitemia and underlying disease, which can be decisive for the treatment period. In the follow-up, this patient never recovered from AA, and died of neutropenic enterocolitis.

For P3, a less severe AA case with a low number of parasites (Eq/mL), allopurinol was prescribed as an alternative antiparasitic therapy. Temporary suppression of parasitemia was observed during allopurinol, but PCR returned to positive 18 months later, after the end of this therapy (Table 1). Remarkably, it reverted to negative 10 months later, after danazol-induced hematological improvement, accompanied by increased numbers of red blood cells and megakaryocytes. Conventional PCR remained negative until the end of follow-up, 23 months after danazol interruption. The pivotal role of danazol in increasing the low levels of Treg cells reported in AA patients [5] perhaps contributes to restoring T lymphocyte and macrophage functions, the main effector cell to control parasite growth and revert PCR to negative. In addition, experimental infections in mice have shown the crucial role of myeloid-derived suppressor cells in controlling inflammation during acute Chagas disease [26]. After “in vivo” depletion of these cells increased secretion of cytokines (IL-6, IFN-γ) and Th17 response, high parasitemia and mortality were observed.

Our three cases added new information to the previously reported *T. cruzi* infected AA patient [6]. This patient received hematopoietic stem cells from a *T. cruzi* uninfected donor, had GVHD and CDR after the transplant, and recovered after 60 days of benznidazole treatment [6].

For the management of AA patients, we should consider, on the one hand, the intensity of hypocellularity and the myelotoxicity of antiparasitic drugs, and, on the other hand, the low parasitemia in our severe AA patients and the report of CDR in one case after a regimen of severe immunosuppression after transplantation [6].

In addition, it is necessary to keep in mind that the antiparasitic treatment aims to either cure (especially in the acute phase and CDR), decrease parasitemia, or interfere with the evolution of Chagas disease [8].

Considering these facts, preemptive therapy seems to be a more reliable recommendation for AA patients without CDR in different situations:

1. Patients with parasitemia (cPCR or blood culture/xenodiagnosis) under a highly immunosuppressive regime (ATG or HSCT transplantation) or only high parasitemia ≥ 100 parasites Eq/mL by qPCR under another immunosuppressive regimen. In addition, because of the known myelotoxicity of more efficient antiparasitic drugs, a shorter regimen of benznidazole could be an option based on promising studies [27], at 300 mg/d for four weeks until new alternatives can be offered.
2. Patients with a low number of parasites (Eq/mL) by qPCR, parasitemia by cPCR, or any parasitological enrichment method persistently positive under less aggressive therapy, should be carefully considered as benznidazole poses a greater risk of granulocytopenia worsening in patients whose parasitemia is under control. For this situation, a safer option is represented by less toxic schemes such as intermittent benznidazole, whose preliminary results need to be confirmed [28], or allopurinol. Although other studies have shown partial success with allopurinol [23,24,25], only a suppressive transitory effect on P3′s parasitemia was observed, corroborating a recent heart transplantation review [29]. Other combinations of synergistic drugs successfully tested in restricted systems should be expanded on in further studies [30].

Alongside the search for new effective and less toxic drugs or drug combinations for antiparasitic treatment, the role of different cells and innate immunity, as well as the role of immunosuppressive drugs such as danazol in the control of *T. cruzi* parasitemia, continue to be challenges to be addressed in other studies.

In summary, we observed that the monitoring of parasitemia by cPCR/qPCR was proven to be a useful tool for the introduction of a preemptive antiparasitic therapy in two patients, who had a conversion of parasitemia from positive to negative temporarily or until the end of follow-up. The same application of molecular methods has been previously reported, in the coinfection *T. cruzi*-HIV or other immunosuppressive conditions [12,13,14,15]. Regarding the most efficient but potentially toxic antiparasitic drugs, our patient received BNZ for 50 days, but to prevent toxicity shorter regimens have been considered [27]. Furthermore, in the case of aggressive immunotherapy such as ATG in patients with AA and *T. cruzi* parasitemia, shorter and less toxic schemes are options to be prescribed under strict control of myelotoxicity and parasitemia, which are deserving of future studies.

## Figures and Tables

**Table 1:** Follow-up of one patient with aplastic anemia and chronic Chagas disease (P1) according to hematological parameters, parasitemia, antiparasitic (benznidazole), and immunosuppressive and antiparasitic therapies.

Patient/Age/Sex/Clinical Form	Myelogram and Blood Count					Therapy		*T. cruzi* Parasitemia				Outcome
	Date	Myelogram/BM Biopsy/Transfusion	WBC mm^3^: Granulocyte/Lymphocyte/Monocyte	RBC 10^6^/mm^3^ Hemoglobin g/dL	Platelets/mm^3^	Start-End	Immuno- Suppressor Drug	Collection Date	cPCR ^b^	qPCR ^b^	Parasitological	
P1/54/M	01/30/19	Intense hypocellularity 3 series MΦ present	1970/760/990/220	1.14/4.1	8000							
	02/11/19		1970/780/990/201	1.4/4.1		06/02/19	Cyclosporine					
						03/11/20	No response					
Cardiac AV Block	03/07/19	^a^ twice	1870/510/1120/230	1.85/5.9				03/27/19	P	U	^c^ MN	
	04/20/19		2180/1000/980/170	2.83/8.8		04/08/19	BNZ ^d^ 51 days					
						05/29/19		04/23/19	N	-	^c^ MN	
	04/30/19		2430/340/1880/210	/8.4	5000							
	05/06/19	^a^ 05/07/19	1820/1200/200	2.23/6.8								
	05/20/19	^a^ 05/16/19	2610/350/1910/340	2.42/7.8	43,000							
	05/25/19		1400/330/860/210	/5.7	4000							
	05/28/19		1060/290/590/200-	1.99/5.9	1000	05/29/19 06/02/19	ATG	06/27/19	N		^c^ MN	
	06/02/19		340/270/30/20	2.31/7.1	9000	05/29/19 07/17/19	Corticosteroid					
	06/10/19		880/540/170/170	2.69/8.3	10,000							
	06/26/19	^a^ 06/27/19	2150/1000/800/300	2.2/6.5		up to 03/11/20	Cyclosporine No response					
	07/24/19	^a^ twice	1180/560/440/190	2.32/7.0		06/10/19 10/17/19	Prednisone	08/12/19	N		^c^ MN	
	10/17/19	^a^ 9 times	1190/300/600/200	2.3/6.8	2700							
	12/10/19	BM biopsy Hypocellularity 3 series PlasmocytesC56+	CD4/CD8 = 0.6 (05/12/2020 = 0.18)									
						03/02/20 03/06/20	Alemtuzumab	10/13/20	N		^c^ MN	Neutropenic enterocolitis 07/31/21
	05/11/21	weekly 2020/2021	890/38/827/21	20.2/6.4	2600							
	07/01/21		240/0/200	2.7/7.6	15,900			07/31/21	N			
								08/02/21	N	U	Skin biopsy N	† 08/02/21

CD, Chagas disease. BM, bone marrow. CA, cardiac form. RBC, red blood cells × 10^6^/mm^3^. AV block, atrioventricular block. MΦ, macrophage. MMF, mycophenolate mofetil. (-) Not Reported. cPCR, conventional polymerase chain reaction. qPCR, quantitative PCR. Number of parasites Eq/mL; N = negative, P = positive, U = Not detected; ^a^ RBC and Platelets transfusion; ^b^ qualitative and quantitative; ^c^ M = direct microscopy for *T. cruzi* identification on peripheral blood; ^d^ antiparasitic drugs. Dates, MM/DD/YY. †—death

**Table 2:** Follow-up of two AA patients with chronic Chagas disease (P2, P3), according to hematological parameters, parasitemia, and the immunosuppressive and antiparasitic therapies.

Patient/Age/Sex/ Clinical Form	Myelogram and Blood Count					Therapy		*T. cruzi* Parasitemia				Outcome
	Date	Myelogram/BM Biopsy/Transfusion	WBC mm^3^: Granulocyte/Lymphocyte/Monocyte	RBC 10^6^/mm^3^ Hemoglobin g/dL	Platelets/mm^3^	Start-End	Immuno-Suppressor Drug	Collection Date	cPCR ^a^	qPCR ^a^	Parasitological	
P2/47/F/	05/21/16		1910/450/1230	1.0/3.1	1000	05/20/16	MMF					
	06/02/16	Hypocellularity 3 series Policlonal PlasmocytesC56+	1530/500/900	2.6/7.8	2100	06/08/16 08/10/16	Cyclosporine					
Atypical Cardiop- athy	07/19/16		1840/450/840	2.8/7.8	3000	07/15/16 07/19/16	ATG	07/08/16	N	U	^b^ MN	
						up to 08/25/16	Prednisone	07/20/16	N	U	^b^ MN	
								07/25/16	N	U	^b^ MN	
	09/05/16	Intense Hypocellularity 3 series	620/200/400	2.8/7.4	2400	08/24/16 12/22/16	Cyclosporine	09/05/16 09/26/16	N N	U U	^b^ MN	
	11/03/16		800/230/430	2.1/6.1	7000			11/01/16	N	-	^b^ MN	Neutropenic enterocolitis
								11/29/16	N	-	^b^ MN	
	01/19/17		370/100/300	3.3/9.4	4400	02/01/17	Danazol					
	02/13/17		110	2.2/6.0	0							† 02/13/17
P3/63/F/	12/14/11	Hypocellularity 3 series. Reticulogenesis-grade 2	1900/500/1200	-/6.0	117,000	12/14/11 01/08/13	Cyclosporine					
Indeterminate Form	03/07/12		2710/1100/1400	-/7.3	35,700			03/27/12	P	9.3	^b^ MN	
	06/15/12		3350/1600/1400	-/9.6	86,000			06/04/12	P	U	^c^ CP/XN	
								09/18/12	P	4.5	^c^ CP/XN	
	03/19/13		2530/1200/1100	-/8.3	97,000	01/09/13-03/18/15	Danazol	03/12/13	P	1,2	^c^ CN/XP/^c^ MN	
						05/23/13-06/30/13	Allopurinol *^d^*	06/18/13	N	U	^c^ CN/XN/^c^ MN	
	10/09/13		4140/2300/1300	-/10.0	207,000			10/15/13	P	0.16	^c^ CN/^c^ MN	
	03/28/14		4150/2490/1300	-/11.3	200,000			03/28/14	P	U	^c^ CN	
	01/21/15		4270/2200/1500	-/10.8	169,000			01/12/15	P	U	^c^ MN	
	11/18/15		3080/1690/1130	-/10.2	89,000			11/23/15	N	U	^b^ MN	
	05/24/16		2940/1250/1350	-/10.1	101,000			05/24/15	N	U	^b^ MN	
	09/02/16		2660/1120/1240	-/10.1	122,000			09/05/16	N	U	^b^ MN	Alive 02/14/17
	02/14/17		3350/1530/1340	-/11.0	126,000			02/06/17	N	-	serology +	

CD, Chagas disease. BM, bone marrow. CA, cardiac form. RBC, red blood cells × 10^6^/mm^3^. AV block, atrioventricular block. MΦ, macrophage. MMF, mycophenolate mofetil. (-) Not Reported. cPCR, conventional polymerase chain reaction. qPCR, quantitative PCR. Number of parasites Eq/mL; N= negative, P= positive, U= not detected; ^a^ qualitative, quantitative; ^b^ M = direct microscopy for *T. cruzi* identification on peripheral blood; ^c^ C= blood culture, and X = xenodiagnosis; (^d^) antiparasitic drugs. Dates, MM/DD/YY, †—death.

## Data Availability

Not applicable.

## References

[1] Liu C., Sun Y., Shao Z. (2019). Current Concepts of the Pathogenesis of Aplastic Anemia. Curr. Pharm. Des..

[2] Young N.S., Calado R.T., Scheinberg P. (2006). Current concepts in the pathophysiology and treatment of aplastic anemia. Blood.

[3] Shinohara K. (2012). The immune-mediated pathogenesis and treatment of aplastic anemia. Bull. Yamaguchi Med. Sch..

[4] Peffault de Latour R., Kulasekararaj A., Iacobelli S., Terwel S.R., Cook R., Griffin M., Halkes C.J.M., Recher C., Barraco F., Forcade E. (2022). For the severe aplastic anemia working party of the European Society for Blood and Marrow Transplantation. Eltrombopag is added to immunosuppression in severe aplastic anemia. N. Engl. J. Med..

[5] Khurana H., Malhotra P., Sachdeva M.U., Varma N., Bose P., Yanamandra U., Varma S., Khadwal A., Lad D., Prakash G. (2018). Danazol increases T regulatory cells in patients with aplastic anemia. Hematology.

[6] Altclas J., Sinagra A., Dictar M., Luna C., Veron M.T., De Risio A.M., García M.M., Salgueira C., Riarte A. (2005). Chagas disease in bone marrow transplantation: An approach to preemptive therapy. Bone Marrow Transplant..

[7] World Health Organization (2015). Chagas disease in Latin America: An epidemiological update based on 2010 estimates. Wkly. Epidemiol. Rec..

[8] Dias J.C.P., Ramos A.N., Gontijo E.D., Luquetti A., Shikanai-Yasuda M.A., Coura J.R., Torres R.M., Melo J.R.C., Almeida E.R., Oliveira W. (2016). 2nd Brazilian Consensus on Chagas Disease, 2015. Rev. Soc. Bras. Med. Trop..

[9] Sartori A.M., Ibrahim K.Y., Nunes Westphalen E.V., Braz L.M., Oliveira O.C., Gakiya E., Lopes M.H., Shikanai-Yasuda M.A. (2007). Manifestations of Chagas disease (American trypanosomiasis) in patients with HIV/AIDS. Ann. Trop. Med. Parasitol..

[10] Shikanai-Yasuda M.A., Mediano M.F.F., Novaes C.T.G., Sousa A.S., Sartori A.M.C., Santana R.C., Correia D., Castro C.N., Severo M.M.S., Hasslocher-Moreno A.M. (2021). Clinical profile and mortality in patients with *T. cruzi*/HIV co-infection from the multicenter data base of the “Network for healthcare and study of *Trypanosoma cruzi*/HIV coinfection and other immunosuppression conditions”. PLoS Negl. Trop. Dis..

[11] De Freitas V.L.T., da Silva S.C., Sartori A.M., Bezerra R.C., Westphalen E.V., Molina T.D., Teixeira A.R.L., Ibrahim K.Y., Shikanai-Yasuda M.A. (2011). Real time PCR in HIV/*Trypanosoma cruzi* coinfection with and without Chagas disease reactivation: Association with HIV viral load and CD4 level. PLoS Negl. Trop. Dis..

[12] Reimer-McAfee M.J., Mejia C., Clark T., Terle J., Pajuelo M.J., Cabeza J., Lora M.H., Valencia E., Castro R., Lozano D. (2021). An Evaluation of the Use of Real-Time Quantitative Polymerase Chain Reaction to Measure Levels of *Trypanosoma cruzi* Parasitemia in HIV Patients in Cochabamba, Bolivia. Am. J. Trop. Med. Hyg..

[13] Sánchez A.G., Baenas D.F., Bonisconti F., Salinas M.J.H., Alvarellos A., Saurit V., Caeiro F., Salomone O., Caeiro J.P., Carballo J. (2021). Reactivation of Chagas Disease in Patients with Rheumatic Autoimmune Diseases Diagnosed by Molecular Quantification Techniques. Clin. Rheumatol..

[14] Cura C.I., Lattes R., Nagel C., Gimenez M.J., Blanes M., Calabuig E., Iranzo A., Barcan L.A., Anders M., Schijman A.G. (2013). Early molecular diagnosis of acute Chagas disease after transplantation with organs from *Trypanosoma cruzi*-infected donors. Am. J. Transplant..

[15] Balderramo D., Bonisconti F., Alcaraz A., Giordano E., Sánchez A., Barrabino M., Caeiro J.P., Alvarellos T., Maraschio M. (2017). Chagas disease and liver transplantation: Experience in Argentina using real-time quantitative PCR for early detection and treatment. Transpl. Infect. Dis..

[16] Wirth J.J., Kierszenbaum F., Sonnenfeld G., Zlotnik A. (1985). Enhancing effects of gamma interferon on phagocytic cell association and killing of *Trypanosoma cruzi*. Infect. Immun..

[17] Reed S. (1988). In vivo administration of recombinant interferon gamma induces macrophage activation, and prevents acute disease, immune suppression, and death in experimental *Trypanosoma cruzi* infections. J. Immunol..

[18] Hoft D.F., Schnapp A.R., Eickhoff C.S., Roodman S.T. (2000). Involvement of CD41 Th1 cells in systemic immunity protective against primary and secondary challenges with *Trypanosoma cruzi*. Infect. Immun..

[19] Carlier Y., Truyens C. (2015). Congenital Chagas disease as an ecological model of interactions between *Trypanosoma cruzi* parasites, pregnant women, placenta and fetuses. Acta Trop..

[20] Weinberger M., Elatter I., Marshall D., Steinberg S.M., Redner R.L., Young N.S., Pizzo P.A. (1992). Patterns of infections in patients with aplastic anemia and the emergence of *Aspergillus* as a major cause of death. Medicine.

[21] Torres H.A., Bodey G.P., Rolston K.V.I., Kantarjian H.M., IRaad I.I., Kontoyiannis D.P. (2003). Infections in patients with aplastic anemia. Cancer.

[22] Lertpongpiroon R., Rattarittamrong E., Rattanathammethee T., Chai-Adisaksopha C., Tantiworawit A., Salee P., Norasetthada L. (2018). Infections in patients with aplastic Anemia in Chiang Mai University. BMC Hematol..

[23] Almeida D.R., Carvalho A.C., Branco J.N., Pereira A.P., Correa L., Vianna P.V., Buffolo E., Martinez E.E. (1996). Chagas’ disease reactivation after heart transplantation: Efficacy of allopurinol treatment. Heart Lung Transplant..

[24] Zulantay I., Honores P., Solari A., Apt W., Ortiz S., Osuna A., Rojas A., López B., Sánchez G. (2004). Use of polymerase chain reaction (PCR) and hybridization assays to detect *Trypanosoma cruzi* in chronic chagasic patients treated with itraconazole or allopurinol. Diagn. Microbiol. Infect. Dis..

[25] Apt W., Arribada A., Zulantay I., Solari A., Sanchez G., Mundaca K., Coronado X., Rodríguez J., Gil L.C., Osuna A. (2005). Itraconazole or allopurinol in the treatment of chronic American trypanosomiasis: The results of clinical and parasitological examinations 11 years post-treatment. Ann. Trop. Med. Parasit..

[26] Arocena A.R., Onofrio L.I., Pellegrini A.V., Silva A.E.C., Augusto Paroli A., Cano R.C., Aoki M.P., Gea S. (2014). Myeloid-derived suppressor cells are key players in the resolution of inflammation during a model of acute infection. Eur. J. Immunol..

[27] Torrico F., Gascón J., Barreira F., Blum B., Almeida I.C., Alonso-Veja D., Barboza T., Bilbe G., Correia E., Garcia W. (2021). New regimens of benznidazole monotherapy and in combination with fosravuconazole for treatment of Chagas disease (BENDITA): A phase 2, double-blind, randomised trial. Lancet Infect. Dis..

[28] Álvarez M.G., Ramirez J.C., Bertocchi G., Fernández M., Hernández Y., Lococo B., Constanza Lopez-Albizu C., Schijman A., Cura C., Abril M. (2020). New scheme of intermitent benznidazole administration in patients chronically infected with *Trypanosoma cruzi*: Clinical, parasitological and serological assessment after three years of follow up. Antimicrob. Agents Chemother..

[29] Nogueira S.S., Felizardo A.A., Caldas I.S., Gonçalves R.V., Novaes R.D. (2018). Challenges of immunosuppressive and antitrypanosomal drug therapy after heart transplantation in patients with chronic Chagas disease: A systematic review of clinical recommendations. Transplant. Rev..

[30] Mazzeti A.L., Capelari-Oliveira P., Bahia M.T., Mosqueira V.C.F. (2021). Review on Experimental Treatment Strategies Against *Trypanosoma cruzi*. J. Exp. Pharmacol..

